# Does Asymmetric Reproductive Isolation Predict the Direction of Introgression in Plants?

**DOI:** 10.3390/genes16020124

**Published:** 2025-01-23

**Authors:** Noland H. Martin, Alexander S. Zalmat, Bailey S. Ellis, Sophia McGarvey, Kayla Simmons-Frazier, Katelin Mancusi, V. Alex Sotola

**Affiliations:** 1 Department of Biology, Texas State University, San Marcos, TX 78666, USA; 2 Biology Department, SUNY Oneonta, Oneonta, NY 13820, USAvasotola@oneonta.edu (V.A.S.)

**Keywords:** asymmetric reproductive isolation, asymmetric introgression, natural hybridization, gene flow, hybrid zones, plant speciation

## Abstract

The evolution of reproductive isolation (RI) results in the reduction of interspecific hybridization and the maintenance of species boundaries. Asymmetries in RI, where one species more frequently serves as the maternal or paternal parent in initial F_1_ hybrid formation, are commonly observed in plants. Asymmetric introgression, the predominantly unidirectional transfer of genetic material through hybridization and backcrossing, has also been frequently documented in hybridizing plant taxa as well. This study investigates whether asymmetries in total RI measured between species can predict the direction of introgression in naturally hybridizing plant taxa. A meta-analysis was conducted on 19 plant species pairs with published data on both asymmetric total RI, and asymmetric introgression. Species pairs that met these criteria were identified through a comprehensive literature review. A two-tailed binomial test was performed to evaluate whether asymmetric RI was associated with asymmetries in introgression. No significant relationship was found between asymmetries in total RI and the direction of introgression (*p* = 0.3593). Asymmetric RI largely does not predict the direction of introgression. Rather, introgression patterns may be better understood by examining F_1_ and later-generation hybrids in natural settings, focusing on their fitness, mating behaviors, and the ecological and demographic factors that shape hybrid zones.

## 1. Introduction

Speciation is a fundamental evolutionary process characterized by the development of reproductive isolation (RI), which limits gene flow between genetically diverging taxa. The total RI observed between taxa typically encompasses a diverse suite of reproductive barriers that act collectively to limit gene flow and maintain species boundaries [1,2,3]. These barriers are broadly categorized based on the timing at which they occur during the life cycle of the organisms. Prezygotic barriers, which act prior to fertilization, reduce the likelihood of F_1_ hybrid formation (e.g., temporal and ecological isolating barriers) [4], whereas postzygotic barriers act after fertilization, manifesting as reduced hybrid viability and/or fertility [3,5]. According to the biological species concept, speciation is complete when RI prevents the production of fertile hybrids, thereby halting gene flow entirely [2].

Complete RI rarely evolves instantaneously (but see [6,7,8]), and the total RI observed between diverging taxa, which can vary across both time and space [9] is often incomplete (i.e., RI ≠ 1.0) allowing for F_1_ and later-generation hybrid formation. Hybridization can have a number of evolutionary consequences ranging from the fusion of hybridizing taxa (particularly when RI is weak) to the transfer of adaptive traits (even when RI is strong), or even the production of new reproductively isolated taxa [3,4]. A number of methods have been developed to quantify RI for individual reproductive barriers and their relative contributions to the total RI observed between hybridizing species. These methods generally seek to quantify the degree to which F_1_ hybrid formation is reduced relative to that of pure-species formation [5,10,11,12,13,14]. Because initial F_1_ hybrid formation can occur bi-directionally (i.e., either species may serve as the maternal or paternal parent), measures of RI are often calculated reciprocally [12,13,15,16]. A key finding across a broad suite of plants is that total RI is frequently asymmetric, with one species more likely to serve as the maternal or paternal parent during initial F_1_ hybrid formation [15,16]. Such asymmetric isolation has led researchers to suggest that this could influence patterns of introgression–the transfer of genetic material between species via hybridization and subsequent backcrossing [17,18]. Like RI, introgression is also often observed to be asymmetric, with gene flow predominantly occurring from one species into the other [17,19,20,21,22]. However, it remains an open question whether asymmetries in RI are predictive of the direction of introgression.

Asymmetries in RI have been widely documented in plants and may result from a combination of sequentially acting prezygotic and postzygotic barriers that may ultimately favor one parent species over the other during F_1_ hybrid formation [13,15,16]. Similarly, asymmetric introgression is also a frequently observed phenomenon in plants [17,19,20,21,22]. Some studies have posited that the directionality in RI might be indicative of the directionality of introgression, often assuming that the favored paternal parent in F_1_ hybrid formation will also serve as the primary genetic donor in subsequent gene flow [17,18].

The direction of introgression is influenced by multiple factors, the interactions of which can be complex [17]. The initial proximity of F_1_ hybrids to one or the other species may play a crucial role, particularly in plants, where pollen and seed dispersal mechanisms can bias backcrossing toward either the most abundant or geographically closest species [22,23,24]. After viable and fertile F_1_ hybrids are formed, selection pressures on later-generation backcross hybrids, whether ecological or intrinsic, can influence the direction of introgression, as selectively advantageous alleles can be incorporated into heterospecific genomic backgrounds [17,25,26,27,28]. Importantly, the predominant direction of introgression may not necessarily be determined by the direction of initial F_1_ hybrid formation, but rather the fitness and mating patterns of those F_1_ and later-generation backcross hybrids. This highlights the need for empirical studies that consider not only RI asymmetries, but also the other factors that may predict the direction of introgression once F_1_ hybrids are formed in natural populations.

This study seeks to clarify the relationship between asymmetric total RI and asymmetric introgression in plants. While numerous studies have documented asymmetric RI in plant taxa [5,15,16], and others have observed asymmetric introgression [17,19,20,21,22], no comprehensive effort has yet been made to synthesize these findings and determine whether asymmetries in RI are predictive of the directionality of introgression. Given the prevalence of hybridization and the recognized evolutionary significance of introgression in plants, understanding whether asymmetries in RI are predictive of the direction of introgression could enhance the understanding of speciation in the face of gene flow and improve the ability to anticipate patterns of gene flow and species integrity in hybridizing taxa.

This review focuses exclusively on plants due to the extensive documentation of hybridization and introgression across a diversity of taxa, as well an extensive body of literature providing quantitative measures of prezygotic, postzygotic, and total RI [5,15,16]. Plant systems are particularly suitable for studying these dynamics because many species readily hybridize, and reproductive barriers in plants are often characterized by a combination of ecological and genetic factors [29]. Additionally, the relatively large sample size of available plant studies allows for robust meta-analyses and the identification of general patterns across taxa. By examining plant species with documented asymmetries in both RI and introgression, this review aims to test whether the direction of RI is predictive of the directionality of introgression.

A meta-analysis approach was utilized to address two main objectives: (1) to identify plant-species pairs where *both* total RI and asymmetries in introgression have been documented (either within the same publication or across separate studies) and (2) to test whether asymmetric total RI is predictive of the direction of introgression. These findings ultimately suggest that asymmetrical RI is not predictive of introgression directionality, highlighting the need for empirical studies on introgression patterns in natural populations to avoid oversimplified assumptions based solely on RI asymmetry.

## 2. Methods and Analysis

For this meta-analysis, we identified naturally hybridizing plant species (i.e., taxa designated as distinct species by the original authors, and not “ecotypes” or other sub-species designations) using a two-step process. First, studies of species pairs must have included at least one measure of prezygotic isolation, at least one measure of postzygotic isolation, and bi-directional calculations of total isolation all reported within a single manuscript. Second, these same species pairs also needed documented evidence of asymmetric introgression, either within the same manuscript described above or in other publications. The species names presented in this study (including the original describing author[s] when provided [Table S1]), are reported as they originally appear in the cited sources.

Christie et al. (2022) [16] compiled a comprehensive dataset of studies conducted before 15 January 2021 that satisfied the first criterion. Total RI calculations derived from those studies are presented in Table 1 (See Table S1 for calculations of Total RI based on methods by Sobel and Chen (2014) [13]). Additional studies meeting the first criterion and published from January 2021 to 30 May 2024 were also identified by using the *Google Scholar* “cited by” link to the Christie et al. (2022) [16] review, as well as the Lowry et al. (2008) [15] and Baack et al. (2015) [5] reviews. The *Google Scholar* database from 2021 onward (up until 30 May 2024) was additionally searched using combinations of the phrases “reproductive isolation”, “plants”, “prezygotic barriers”, “postzygotic barriers”, “total isolation”, “prezygotic isolation”, and “postzygotic isolation”. These are also presented in Table 1.

Once species pairs meeting criterion 1 were identified, a comprehensive search of *Google Scholar* was again conducted in order to determine whether additional studies were published that examined introgression between the species pairs identified above and, if so, to ascertain any asymmetries with respect to such gene flow. For each species pair, relevant literature was identified by performing a three-word search combining the genus and both specific epithets. This approach was necessary as some authors did not use the full species names for both taxa and may have abbreviated the genus name for one or the other resulting in their studies not appearing in a *Google Scholar* search that utilized the unabbreviated names of both species. The resulting papers were examined to determine whether any asymmetry in gene flow existed. For some species pairs, the initial search produced an unwieldy number of results. For these taxa, the search was further narrowed by incorporating additional combinations of the keywords “introgression”, “gene flow”, and “asymmetric”.

To assess whether asymmetries in total RI were predictive of asymmetries in introgression, a two-tailed binomial test was performed using the *binom.test* function in Program R. For each species pair with identified asymmetries in both total RI and introgression, whether or not those asymmetries were in the same or opposite directions was recorded. For this analysis, a ‘success’ was recorded when RI and introgression occurred in the same direction, and a ‘failure’ was recorded when the direction was in opposite directions. The null hypothesis was that there was no relationship between asymmetries in reproductive isolation and asymmetries in introgression (e.g., the directionality of RI was not predictive of the directionality of introgression; probability of success = 0.5).

## 3. Results and Discussion

This study investigated whether asymmetries in total reproductive isolation (RI) were predictive of the direction of introgression in hybridizing plant taxa. A total of 19 species pairs were identified where published information existed for both total RI and asymmetric introgression (Table 1). A binomial test (N successes = 12, N trials = 19, *p* = 0.3593) showed no significant relationship between directionality of asymmetries in total RI and introgression, suggesting that introgression patterns are instead more often shaped by a combination of system-specific ecological, genetic, evolutionary and/or demographic factors.

In many of the systems identified here (N = 12/19), asymmetry in total RI corresponds with that of introgression, where the species serving primarily as the maternal parent also tends to receive more introgressed genetic material from the species that serves as the pollen parent (Table 1). In a majority of these cases (8 of 12), the direction of introgression appears to be driven primarily by demographic factors such as relative species abundances, range expansions of one species into the habitat of another, or spatial shifts in hybrid zones. For instance, in *Primula*, directional introgression from *P. beesiana* Forrest into *P. bulleyana* Forrest was attributed primarily to a greater abundance of *P. bulleyana* in hybridizing populations, which facilitated increased amounts of pollinator-mediated backcrossing towards *P. bulleyana* [49]. In an *Ipomopsis* hybrid zone, asymmetric introgression from *I. tenuituba* into *I. aggregata* was attributed to *I. aggregata* advancing into *I. tenuituba* habitats facilitated by pollinator behavior and habitat selection on hybrids [37,38,56,57]. Similarly, in *Quercus*, alleles from *Q. mongolica* were found to have introgressed into *Q. liaotungensis*, likely resulting from northward migration of *Q. liaotungensis* into already-colonized *Q. mongolica* habitats during warmer climatic periods [53,54]. Asymmetric introgression in two *Pinus* hybrid zones was attributed to unidirectional pollen flow and historical range shifts influenced by geological and historical climatic changes [47,48]. Similar patterns are also observed in *Iris*, where introgression of chloroplast DNA from *I. innominata* into *I. douglasiana* was attributed to hybrid zone movement [39]. In *Mimulus*, asymmetric introgression from *M. cardinalis* into *M. lewisii* has been reported, though it is unclear if such introgression is due to range expansion or the spread of adaptive alleles via natural selection [41]. Similarly in *M. glaucescens* and *M. guttatus* hybridizing populations, gene flow from *M. glaucescens* into *M. guttatus* was attributed to increased migration rates of the former, though selective costs of introgressed *M. guttatus* alleles into *M. glaucescens* backgrounds were not ruled out [42]. While these studies highlight the importance of demographic factors in shaping introgression patterns, it is important to recognize that such demographic factors are often already incorporated into measures of reproductive isolation. Therefore, any observed correspondence between RI asymmetry and introgression direction could simply be due to chance alone.

The remaining studies where asymmetry in total RI corresponded with the direction of introgression (N = 4 of 12) suggested alternative explanations beyond demographic factors, including natural selection, differences in mating systems, or no clear mechanism for explaining the observed asymmetries. For example, in *Penstamon*, introgression from *P. centranthifolius* into *P. spectabilis* was been observed [45], with significant reductions in seed number and seed mass being observed in backcrosses towards *P. centranthifolius* (but not towards *P. spectabilis*) posited as a possible driver of this asymmetry [46]. Selection on floral traits and mating system differences likely explain asymmetric introgression from the predominantly selfing *Ipomoea lacunosa* into the outcrossing *I. cordatotriloba* [35,36]. In the hybridizing systems involving *Costus pulverulentus* and *C. scaber* [32] and *Primula poissonii* Franchet and *P. secundiflora* Franchet [50], the directionality of asymmetric total RI aligned with introgression patterns, though no clear mechanism for these asymmetries were proposed. While these studies suggest that factors beyond demography can influence introgression patterns, the lack of clear documented mechanistic explanations in some cases underscores the need for more detailed investigations into the specific drivers of introgression in these systems.

The remaining study systems examined showed contrasting patterns (N = 7/19), where asymmetries in total RI and introgression occurred in *opposite* directions. In these cases, the species serving primarily as the maternal parent received gene flow *less* frequently than in the reciprocal direction. For example, in *Centaurium*, total RI favored F_1_ hybrid production with *C. littorale* (D. Turn.) Gilm. as the maternal parent and *C. erythraea* Rafn as the paternal parent (RI*_C. erythraea_* = 0.9986; RI*_C. littorale_* = 0.9879, Table 1). However, introgression occurred predominantly into *C. erythraea*, a pattern that was largely attributed to differences in mating systems, with *C. littoral* exhibiting higher rates of selfing, and F_1_ and late-generation hybrids being more likely to mate with the outcrossing species *C. erythrea* [30,31]. Similarly, asymmetric introgression in *Mimulus* occurred predominantly from the selfing *M. nasutus* into the largely outcrossing *M. guttatus*, this despite total RI being complete when *M. guttatus* acted as the F_1_ pollen parent (RI*_M. guttatus_* = 1.0, Table 1) [12,20]. In protandrous *Silene* species, flowering asynchrony was identified as a primary driver of asymmetric total RI (RI*_S.asclepiadae_* = 0.6850, RI*_S. yunnanensis_* = 0.8397). *Silene asclepiadae* Franchet flowering precedes that of *S. yunnanensis* Franchet, and late-flowering *S. asclepiadae* are more likely to serve as seed parents during F_1_ hybrid formation. However, the flowering times of hybrids are most similar to those of *S. yunnanensis* allowing for more backcrossing and introgression towards this species [55]. In *Primula*, total RI was higher when *P. vulgaris* Huds. was the maternal parent compared to *P. elatior* Hill (RI*_P. vulgaris_* = 0.9678, RI*_P. elatior_* = 0.9068), and similarly higher in comparisons between *P. vulgaris* and *P. veris* L., RI was also higher with *P. vulgaris* as the maternal parent (RI*_P.vulgaris_* = 0.9515, RI*_P.veris_* = 0.7150) [18]. Subsequent genomic analysis revealed likely adaptive mechanisms favoring directional introgression from *P. elatior* and *P. veris* into *P. vulgaris* across multiple hybrid zones, including increased fertility and improved tolerance to iron-rich waterlogged soils [51]. These contrary findings clearly indicate that RI asymmetry alone is a poor predictor of the direction of introgression.

Collectively, these findings highlight the important roles of evolutionary, ecological, and demographic factors in shaping the mating patterns and fitness of F_1_ and later-generation hybrids, which ultimately influence introgression patterns. Studies that measure RI are essential for identifying the key barriers to initial F_1_ hybrid formation, but they offer limited insight into the complex processes that unfold *after* such hybrids are formed. Our findings suggest that the directionality of asymmetric RI does not reliably predict the direction of introgression. Interestingly, reproductive isolating barriers are often referred to synonymously as barriers to gene flow, yet it is important to recognize that these terms are not entirely interchangeable: most measures of RI specifically focus on reductions in F_1_ hybrid formation, while gene flow necessarily occurs *after* F_1_ hybrids are formed (and only if F_1_ hybrids reveal some level of fitness). It is therefore important to integrate studies of both RI and gene flow in order to develop a comprehensive understanding of hybrid zone dynamics.

## Figures and Tables

**Table 1:** Total reproductive isolation measured using methods by Sobel and Chen (2014) [13]. RI_species1_ indicates total RI calculated with species 1—the species with the highest measure of total RI—as the seed parent, and RI_species1_ indicates total RI calculated with species 2 as the seed parent. The predominant direction of asymmetric introgression is indicated in the last column, as well as citations where total RI and introgression directions were obtained.

Species 1	Species 2	RI_species1_	RI_species2_	Introgression Direction
*Centaurium erythraea*	*Centaurium littorale*	0.9986	0.9879	Species 1 [30,31] *
*Costus pulverulentus*	*Costus scaber*	1	0.9994	Species 2 [32,33] *
*Helianthus petiolaris*	*Helianthus annuus*	0.99995	0.99990	Species 1 [34] *
*Ipomoea lacunosa*	*Ipomoea cordatotriloba*	0.8270	0.7043	Species 2 [35,36]
*Ipomopsis tenuituba*	*Ipomopsis aggregata*	0.5959	0.3497	Species 2 [37,38] *
*Iris douglasiana*	*Iris innominata*	1	0.6877	Species 2 [39,40] *
*Mimulus cardinalis*	*Mimulus lewisii*	0.9996	0.9955	Species 2 [11,41] *
*Mimulus glaucescens*	*Mimulus guttatus*	0.632	0.39	Species 2 [42]
*Mimulus guttatus*	*Mimulus nasutus*	1	0.984	Species 1 [12] *
*Ophrys incubacea*	*Ophrys garganica*	1	0.9402	Species 1 [43,44] *
*Penstemon centranthifolius*	*Penstemon spectabilis*	0.9889	0.5283	Species 2 [45,46] *
*Pinus tabuliformis*	*Pinus densata*	0.7821	0.6491	Species 2 [47,48] *
*Pinus yunnanensis*	*Pinus densata*	0.5942	0.4988	Species 2 [47,48] *
*Primula beesiana*	*Primula bulleyana*	1	0.6623	Species 2 [49] *
*Primula secundiflora*	*Primula poissonii*	0.9654	0.6682	Species 2 [50] *
*Primula vulgaris*	*Primula elatior*	0.9678	0.9068	Species 1 [18,51] *
*Primula vulgaris*	*Primula veris*	0.9515	0.7150	Species 1 [51,52] *
*Quercus mongolica*	*Quercus liaotungensis*	0.4076	0.1204	Species 2 [53,54] *
*Silene yunnanensis*	*Silene ascelepiadae*	0.8397	0.6850	Species 1 [55] *

* Total isolation derived from RI measures reported by Christie et al. (2022) [16]—See Table S1.

## Data Availability

All data utilized for this study are listed in Table 1 and Table S1.

## Supplementary Materials

The following supporting information can be downloaded at: https://www.mdpi.com/article/10.3390/genes16020124/s1, Table S1: Calculations of Total Isolation.

## References

[1] Dobzhasnky T. (1937). Genetic Nature of Species Differences. Am. Nat..

[2] Mayr E. (1942). Systematics and the Origin of Species from the Viewpoint of a Zoologist.

[3] Orr H.A., Coyne J.A. (2004). Speciation.

[4] Sobel J.M., Chen G.F., Watt L.R., Schemske D.W. (2010). The Biology of Speciation. Evolution.

[5] Baack E., Melo M.C., Rieseberg L.H., Ortiz-Barrientos D. (2015). The Origins of Reproductive Isolation in Plants. New Phytol..

[6] Grant V. (1981). Plant Speciation.

[7] Levin D.A. (2002). The Role of Chromosomal Change in Plant Evolution.

[8] Wood T.E., Takebayashi N., Barker M.S., Mayrose I., Greenspoon P.B., Rieseberg L.H. (2009). The Frequency of Polyploid Speciation in Vascular Plants. Proc. Natl. Acad. Sci. USA.

[9] Arnold M.L., Martin N.H. (2010). Hybrid fitness across time and habitats. Trends Ecol. Evol..

[10] Coyne J.A., Orr H.A. (1989). The Genetics of Postzygotic Isolation in the e H. Allen Orr and Jerry A. Coyne Drosophila Vidis Group. Genet. Soc. Am..

[11] Ramsey J., Bradshaw H.D., Schemske D.W. (2003). Components of Reproductive Isolation between the Monkeyflowers *Mimulus lewisii* and *M. cardinalis* (Phrymaceae). Evolution.

[12] Martin N.H., Willis J.H. (2007). Ecological Divergence Associated with Mating System Causes Nearly Complete Reproductive Isolation between Sympatric *Mimulus* Species. Evolution.

[13] Sobel J.M., Chen G.F. (2014). Unification of Methods for Estimating the Strength of Reproductive Isolation. Evolution.

[14] Stankowski S., Ravinet M. (2021). Defining the Speciation Continuum. Evolution.

[15] Lowry D.B., Modliszewski J.L., Wright K.M., Wu C.A., Willis J.H. (2008). Review. The Strength and Genetic Basis of Reproductive Isolating Barriers in Flowering Plants. Philos. Trans. R. Soc. B Biol. Sci..

[16] Christie K., Fraser L.S., Lowry D.B. (2022). The Strength of Reproductive Isolating Barriers in Seed Plants: Insights from Studies Quantifying Premating and Postmating Reproductive Barriers over the Past 15 Years. Evolution.

[17] Arnold M.L., Tang S., Knapp S.J., Martin N.H. (2010). Asymmetric Introgressive Hybridization among Louisiana Iris Species. Genes.

[18] Keller B., de Vos J.M., Schmidt-Lebuhn A.N., Thomson J.D., Conti E. (2016). Both Morph- and Species-Dependent Asymmetries Affect Reproductive Barriers between Heterostylous Species. Ecol. Evol..

[19] Broyles S.B. (2002). Hybrid Bridges to Gene Flow: A Case Study in Milkweeds (*Asclepias*). Evolution.

[20] Sweigart A.L., Willis J.H. (2003). Patterns of Nucleotide Diversity in Two Species of *Mimulus* Are Affected by Mating System and Asymmetric Introgression. Evolution.

[21] Petit R.J., Bodénès C., Ducousso A., Roussel G., Kremer A. (2004). Hybridization as a Mechanism of Invasion in Oaks. New Phytol..

[22] Currat M., Ruedi M., Petit R.J., Excoffier L. (2008). The Hidden Side of Invasions: Massive Introgression by Local Genes. Evolution.

[23] Prentis P.J., White E.M., Radford I.J., Lowe A.J., Clarke A.R. (2007). Can Hybridization Cause Local Extinction: A Case for Demographic Swamping of the Australian Native Senecio *Pinnatifolius* by the Invasive Senecio Madagascariensis?. New Phytol..

[24] Field D.L., Ayre D.J., Whelan R.J., Young A.G. (2008). Relative Frequency of Sympatric Species Influences Rates of Interspecific Hybridization, Seed Production and Seedling Performance in the Uncommon *Eucalyptus aggregata*. J. Ecol..

[25] Keim P., Paige K.N., Whitham T.G., Lark K.G. (1989). Genetic Analysis of an Interspecific Hybrid Swarm of Populus: Occurrence of Unidirectional Introgression. Genetics.

[26] Cruzan M.B., Arnold M.L. (1994). Assortative Mating and Natural Selection in an Iris Hybrid Zone. Evolution.

[27] Martin N.H., Bouck A.C., Arnold M.L. (2006). Detecting Adaptive Trait Introgression between *Iris fulva* and *I. brevicaulis* in Highly Selective Field Conditions. Genetics.

[28] Suarez-Gonzalez A., Lexer C., Cronk Q.C.B. (2018). Adaptive Introgression: A Plant Perspective. Biol. Lett..

[29] Arnold M.L. (1997). Natural Hybridization and Evolution.

[30] Brys R., Vanden Broeck A., Mergeay J., Jacquemyn H. (2014). The Contribution of Mating System Variation to Reproductive Isolation in Two Closely Related *Centaurium* Species (Gentianaceae) with a Generalized Flower Morphology. Evolution.

[31] Brys R., van Cauwenberghe J., Jacquemyn H. (2016). The Importance of Autonomous Selfing in Preventing Hybridization in Three Closely Related Plant Species. J. Ecol..

[32] Surget-Groba Y., Kay K.M. (2013). Restricted Gene Flow within and between Rapidly Diverging Neotropical Plant Species. Mol. Ecol..

[33] Kay K.M. (2006). Reproductive Isolation Between Two Closely Related Hummingbird-Pollinated Neotropical Gingers. Evolution.

[34] Sambatti J.B.M., Strasburg J.L., Ortiz-Barrientos D., Baack E.J., Rieseberg L.H. (2012). Reconciling Extremely Strong Barriers with High Levels of Gene Exchange in Annual Sunflowers. Evolution.

[35] Rifkin J.L., Castillo A.S., Liao I.T., Rausher M.D., Rifkin J.L., Castillo A.S., Liao I.T., Rausher M.D. (2019). Gene Flow, Divergent Selection and Resistance to Introgression in Two Species of Morning Glories (Ipomoea). Mol. Ecol..

[36] Rifkin J.L., Ostevik K.L., Rausher M.D. (2023). Complex Cross-Incompatibility in Morning Glories Is Consistent with a Role for Mating System in Plant Speciation. Evolution.

[37] Campbell D.R., Waser N.M. (2001). Genotype-by-Environment Interaction and the Fitness of Plant Hybrids in the Wild. Evolution.

[38] Wu C.A., Campbell D.R. (2005). Cytoplasmic and Nuclear Markers Reveal Contrasting Patterns of Spatial Genetic Structure in a Natural *Ipomopsis* Hybrid Zone. Mol. Ecol..

[39] Young N.D. (1996). An Analysis of the Causes of Genetic Isolation in Two Pacific Coast Iris Hybrid Zones. Can. J. Bot..

[40] Young N.D. (1996). Concordance and Discordance: A Tale of Two Hybrid Zones in the Pacific Coast Irises (Iridaceae). Am. J. Bot..

[41] Nelson T.C., Stathos A.M., Vanderpool D.D., Finseth F.R., Yuan Y.W., Fishman L. (2021). Ancient and Recent Introgression Shape the Evolutionary History of Pollinator Adaptation and Speciation in a Model Monkeyflower Radiation (*Mimulus* Section Erythranthe). PLoS Genet..

[42] Ivey C.T., Habecker N.M., Bergmann J.P., Ewald J., Frayer M.E., Coughlan J.M. (2023). Weak Reproductive Isolation and Extensive Gene Flow between *Mimulus glaucescens* and *M. guttatus* in Northern California. Evolution.

[43] Sedeek K.E.M., Scopece G., Staedler Y.M., Schönenberger J., Cozzolino S., Schiestl F.P., Schlüter P.M. (2014). Genic Rather than Genome-Wide Differences between Sexually Deceptive *Ophrys* Orchids with Different Pollinators. Mol. Ecol..

[44] Soliva M., Widmer A. (2003). Gene Flow across Species Boundaries in Sympatric, Sexually Deceptive *Ophrys* (Orchidaceae) Species. Evolution.

[45] Wolfe A.D., Elisens W.J. (1994). Nuclear Ribosomal DNA Restriction-Site Variation in *Penstemon* Section Peltanthera (Scrophulariaceae): An Evaluation of Diploid Hybrid Speciation and Evidence for Introgression. Am. J. Bot..

[46] Chari J., Wilson P. (2001). Factors Limiting Hybridization between *Penstemon spectabilis* and *Penstemon centranthifolius*. Can. J. Bot..

[47] Zhao W., Meng J., Wang B., Zhang L., Xu Y., Zeng Q.Y., Li Y., Mao J.F., Wang X.R. (2014). Weak Crossability Barrier but Strong Juvenile Selection Supports Ecological Speciation of the Hybrid Pine *Pinus densata* on the Tibetan Plateau. Evolution.

[48] Wang B., Mao J.F., Gao J.I.E., Zhao W.E.I., Wang X.R. (2011). Colonization of the Tibetan Plateau by the Homoploid Hybrid Pine *Pinus densata*. Mol. Ecol..

[49] Ma Y.-P., Tian X.-L., Zhang J.-L., Zhi-Kun Wu W., Sun E.-B. (2014). Evidence for Natural Hybridization between *Primula beesiana* and *P. bulleyana*, Two Heterostylous Primroses in NW Yunnan, China. J. Syst. Evol..

[50] Xie Y., Zhu X., Ma Y., Zhao J., Li L., Li Q. (2017). Natural Hybridization and Reproductive Isolation between Two *Primula* Species. J. Integr. Plant Biol..

[51] Stubbs R.L., Theodoridis S., Mora-Carrera E., Keller B., Potente G., Yousefi N., Jay P., Léveillé-Bourret É., Choudhury R.R., Celep F. (2024). The Genomes of Darwin’s Primroses Reveal Chromosome-Scale Adaptive Introgression and Differential Permeability of Species Boundaries. New Phytol..

[52] Keller B., Ganz R., Mora-Carrera E., Nowak M.D., Theodoridis S., Koutroumpa K., Conti E. (2021). Asymmetries of Reproductive Isolation Are Reflected in Directionalities of Hybridization: Integrative Evidence on the Complexity of Species Boundaries. New Phytol..

[53] Liao W.J., Zhu B.R., Li Y.F., Li X.M., Zeng Y.F., Zhang D.Y. (2019). A Comparison of Reproductive Isolation between Two Closely Related Oak Species in Zones of Recent and Ancient Secondary Contact. BMC Evol. Biol..

[54] Zeng Y.F., Liao W.J., Petit R.J., Zhang D.Y. (2011). Geographic Variation in the Structure of Oak Hybrid Zones Provides Insights into the Dynamics of Speciation. Mol. Ecol..

[55] Zhang J.J., Montgomery B.R., Huang S.Q. (2016). Evidence for Asymmetrical Hybridization despite Pre- and Post-Pollination Reproductive Barriers between Two *Silene* Species. AoB Plants.

[56] Campbell D.R., Waser N.M., Wolf P.G. (1998). Pollen Transfer by Natural Hybrids and Parental Species in an *Ipomopsis* Hybrid Zone. Evolution.

[57] Campbell D.R., Crawford M., Brody A.K., Forbis T.A. (2002). Resistance to Pre-Dispersal Seed Predators in a Natural Hybrid Zone. Oecologia.

