# Supplementary material for: Does Asymmetric Reproductive Isolation Predict the Direction of Introgression in Plants?
Source: Genes (Basel). 2025 Jan 23;16(2):124. doi: 10.3390/genes16020124 (PMC11855407; doi:10.3390/genes16020124)
Supplement: Supplementary file 1 [file genes-16-00124-s001.zip › Table_S1_Description.docx.pdf]

Supplementary Table 1 (Table S1):

Bi-directional Calculations of Total Reproductive Isolation

Studies where multiple calculations of the strength of individual reproductive isolating barriers for species pairs are listed in Columns A and B (authors and citation numbers, respectively). Species pairs for which Total Reproductive Isolation was calculated are listed in Columns C-D. Columns E through AF contain measures of individual reproductive isolating barriers taken directly from Supplementary Table S2 and described in Christie et al. (2022) [16], with any exceptions noted and described in column AI ("NOTES"). Columns AC and AD are calculated mean measures of reproductive isolation due to pollen sterility and ovule inviability (as suggested by Christie et al. (2022) in supplemental documentation).

Columns E through J represent prezygotic barriers affecting co-occurrence and are colored orange. Columns K through P represent those prezygotic barriers that do not affect co-occurrence and are colored yellow. Columns Q through AF represent postzygotic barriers and are colored green. Note that columns Y through AB were not directly used for calculating total RI. Instead these numbers were used to calculate average reproductive isolation across pollen sterility and ovule inviability (Columns AC and AD).

Total Reproductive Isolation was calculated separately using Equation $RI_{4E}$ from Sobel and Chen (2014) for both directions of possible $F_1$ hybrid formation and are listed in Columns AG and AH. These calculations were made using the "total isolation" tab in the supplementary worksheet (evo12362-sup-0003-SuppMat) provided in Sobel and Chen (2014).
